# Visual Evoked Potential Responses after Photostress in Migraine Patients and Their Correlations with Clinical Features

**DOI:** 10.3390/jcm10050982

**Published:** 2021-03-02

**Authors:** Gianluca Coppola, Cherubino Di Lorenzo, Davide Di Lenola, Mariano Serrao, Francesco Pierelli, Vincenzo Parisi

**Affiliations:** 1 Department of Medico-Surgical Sciences and Biotechnologies, Sapienza University of Rome Polo Pontino, Corso della Repubblica 79, 04100 Latina, Italy; cherub@inwind.it (C.D.L.); davidedilenola88@libero.it (D.D.L.); mariano.serrao@uniroma1.it (M.S.); francesco.pierelli@uniroma1.it (F.P.); 2 IRCCS—Neuromed, Headache Center, Via Atinense 18, 86077 Pozzilli, IS, Italy; 3 IRCCS—Fondazione Bietti, Via Livenza 3, 00198 Rome, Italy; vincenzo.parisi@fondazionebietti.it

**Keywords:** migraine, visual evoked potentials, photostress, cortical dysexcitability, macula

## Abstract

In the past few years, researchers have detected subtle macular vision abnormalities using different psychophysical experimental tasks in patients with migraine. Recording of visual evoked potential (VEP) after photostress (PS) represents an objective way to verify the integrity of the dynamic properties of macular performance after exposure to intense light. VEPs were recorded before and after PS in 51 patients with migraine (19 with aura (MA) and 22 without aura (MO) between attacks, and 10 recorded during an attack (MI)) and 14 healthy volunteers. All study participants were exposed to 30 s of PS through the use of a 200-watt bulb lamp. The P100 implicit time and N75-P100 amplitude of the baseline VEP were compared with those collected every 20 s up to 200 s after PS. VEP parameters recorded at baseline did not differ between groups. In all groups, the VEP recordings exhibited a significant increase in implicit times and a reduction in amplitude at 20 s after the PS. In migraine, the percentage decrease in amplitudes observed at 20 s after photostress was significantly lower than in healthy volunteers, in both MO and MA patients, but not in MI patients. When data for MO and MA patients were combined, the percentage of amplitude change at 20 s was negatively correlated with the number of days that had elapsed since the last migraine attack, and positive correlated with attack frequency. We showed dynamic changes of recovery of VEP after PS depending on the migraine cycle. This finding, in conjunction with those previously attained with other neuromodulatory interventions using VEPs, leads us to argue that migraine-disease-related dysrhythmic thalamocortical activity precludes amplitude suppression by PS.

## 1. Introduction

The visual system is deeply involved in the pathophysiology of migraine. Dysfunctions have been described from the outermost layers of the retina [1] to the visual cortex [2,3] and its associative areas [4,5], passing through its thalamic station [6,7]. The abnormalities described are both functional and structural. By applying different technical approaches, a specific involvement of macula cells and an alteration of color vision in the outer retinal layers by cone photoreceptors for the blue–yellow axis has been proposed for migraine patients [8,9]. Other authors have described abnormalities in color perception, such as for red and blue, in addition to cortical involvement [10,11,12,13]. Nevertheless, there is evidence in favor of an involvement in migraine of a specific macular pathway mediated by a population of photosensitive retinal ganglion cells, which are in direct connection with the thalamus, the trigeminal system, and several associated brain areas [14,15].

One method of studying macular cone function is to check the period of recovery of visual acuity after the retina is dazzled with an ophthalmoscope (photostress, PS) [16,17]. PS was also used to test the recovery of excitability of the visual system by recording visual evoked potentials (VEPs) in response to pattern-reversal stimuli [18]. In healthy subjects, PS induces VEP changes consisting of a delayed P100 implicit time and a decrease in N75-P100 amplitude; a recovery of normal VEP morphology after PS takes approximately 60 s [19]. This method has been used several times to study macular function in patients with maculopathies [20,21,22]. Thus, in this study we investigated macular function by recording serial VEPs after PS (every 20 s) in migraine patients with and without aura between and during attacks, in correlation with clinical variables.

Considering previous evidence obtained with VEPs [2,23], we hypothesized that migraine patients between attacks would have a faster recovery of macular function after macular dazzling compared to healthy volunteers, and that this would be related to the time at which the patient was recorded during the migraine cycle.

## 2. Materials and Methods

### 2.1. Participants

We enrolled a group of 51 consecutive migraine patients attending the headache clinic of Sapienza University of Rome Polo Pontino, of which 22 without aura (MO Group, ICHD-III code 1.1, mean age 29.2 ± 8.2 years) and 19 with aura (MA Group, ICHD-III code 1.2.1.1, typical aura with headache, mean age 30.3 ± 10.2 years) underwent VEP recordings during the interictal period, i.e., attack-free for at least three days before and after the recording sessions (checked by collecting headache diaries, and by telephone or e-mail interviews). Ten patients were accidentally recorded during their typical migraine attack (within the first 6 h) and thus considered as an ictal group (MI Group, ICHD-III code 1.1, mean age 32.7 ± 11.7 years).

Fourteen age- and gender-matched healthy volunteers (HV Group, mean age 29.7 ± 6.1 years) were recruited from among university students and medical professionals and randomly recorded between patients. They had to be free of any apparent medical conditions, as well as personal or family history of migraine or epilepsy, and could not take drugs on a regular basis. In order to exclude hormone-related effects, recordings of female participants were performed outside of menses.

After an ophthalmological evaluation, including best-corrected visual acuity, slit-lamp biomicroscopy, intraocular pressure measurement, and indirect ophthalmoscopy, only patients or HVs with absence of optical media and retinal or optic nerve diseases, and with Snellen best-corrected visual acuity of 10/10, were included in the study. There were no patients with suspected retinal migraine.

For migraine patients, we managed to collect up to two months of headache diaries on the day of the recording session. Patients had to indicate duration of migraine history (years), attack frequency (n/month), attack duration (hours), and number of days passed since the last migraine attack (Table 1).

We excluded patients taking medications on a regular basis (e.g., antidepressants, benzodiazepines, prophylactic migraine drugs, corticosteroids, antibiotics), except for oral contraceptives. We carefully excluded subjects who had sleep deprivation or had ingested alcoholic beverages on the day preceding the recording sessions. Caffeinated beverages were not permitted on the day of recordings. All participants received a complete description of the study and granted their written informed consent. The project was approved by the ethical review board of the Faculty of Medicine, University of Rome, Italy.

### 2.2. Visual Evoked Potentials

Recordings were made according to a previously described method [24]. Briefly, we used a full-field checkerboard visual pattern (contrast 80%, mean luminance 250 cd/m^2^, 2 reversals per second) generated on a computer monitor, with a viewing distance of 114 cm (single check edges subtended a 15’ visual angle). We always visually stimulated the right eye, with the left eye covered by a patch. We positioned the active VEP electrode at Oz and the reference electrode at Fz (10/20 system), with a ground electrode placed on the right forearm. We used a Digitimer^TM^ D360 preamplifier, a Cambridge Electronic Design (CED, Cambridge, UK)^TM^ Power1401 analogue-to-digital converter, and the Signal^TM^ software package version 4.11 (CED Ltd.) for the signal’s analysis. For each participant, we collected 40 consecutive sweeps per block trial (4000 Hz sampling rate), each lasting 500 ms. Thereafter, we applied a 100 Hz low-pass digital filter offline, and we accepted no more than two sweeps discarded per block trial because of artifacts.

The transient VEP components (N75, P100, and N145) were identified according to the ISCEV standard for clinical VEPs [25]. We measured the peak-to-peak amplitude of the N75-P100 complex. All VEP recordings were done in the morning (between 09.00 and 11.00 a.m.) by the same investigator (D.D.L.), who was not involved in the patient’s enrollment phase. All recordings were then anonymized and analyzed offline in a blind manner by one investigator (V.P.).

### 2.3. Procedure

The test of recovery of pattern VEPs after macular bleaching was performed according to a published protocol [21,24]. In brief, the procedure consisted of recording the baseline VEP, bleaching the central retina, and recording the VEPs at predetermined times after bleaching.

We performed 30 s of bleaching of the central retina by means of a circular diffusing surface retroilluminated by a 200 W lamp. The participants fixated the center of the circular surface, with natural pupils, from 20 cm. At the participants’ viewing distance, the bleaching field subtended 6°. Since the retinal luminance during bleaching was 3.58 log photopic trolands, in this procedure we estimated bleaching of approximately 20% of the cone photopigments [26]. During the procedure, the pupil diameter, measured by one investigator using a ruler and a magnifying lens, decreased from the prebleaching value (mean 3.4 ± 0.6 mm in both HVs and patients) to 2.1 ± 0.5 mm in HVs and 2.1 ± 0.4 mm in patients. At 20 s after bleaching, the pupil diameter had already recovered to the prebleaching value in all HVs and patients and did not vary significantly during the time of recording.

Immediately after the end of bleaching, the participant was instructed to fixate the center of the visual pattern (marked by a red dot), and VEP recording was started. Although the participants saw a central scotoma, the red target was detected by all. We acquired 400 VEP sweeps that were offline partitioned in 10 block trials (40 sweeps/block, with each block 20 s in length). The P100 implicit time and N75-P100 amplitude in the 10 post-bleaching VEP recordings were measured. VEP P100 implicit times and N75-P100 amplitudes after PS (20 s) were also normalized to baseline, and are given as the percentage change of the basal recording.

### 2.4. Statistical Analysis

We used Statistica (StatSoft Inc., Tulsa, OK, USA) for Windows (version 8.0) for all analyses.

First, we checked VEP data for normal distribution by applying the Kolmogorov–Smirnov test. A preliminary descriptive analysis showed that some VEP N75-P100 peak-to-peak amplitudes of the 10 blocks had a non-normal distribution. After log transformation, all data reached normal distribution (Kolmogorov–Smirnov test, *p* > 0.21).

We made a repeated measures analysis of variance (ANOVA) taking “time” as the within-subject factor (baseline up to 200 s) and “groups” (HV, MO, MA, MI) as between-subject factors. A Tukey test was used for post hoc analysis. A one-way ANOVA was performed in order to compare the VEP P100 implicit time and the N75-P100 amplitude percentage changes at 20 s of the baseline recording. A Pearson’s correlation test was used to search for correlations among the VEP amplitudes and metric clinical variables. *p* values ≤ 0.05 were considered to indicate statistical significance.

## 3. Results

**Figure 1:**
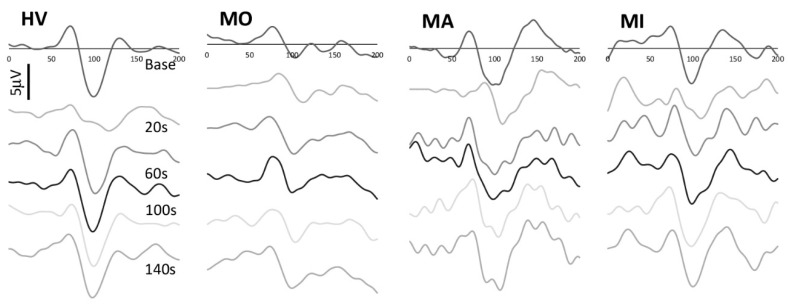
Representative recordings of visual evoked potentials recorded at baseline and at 20, 60, 100, and 140 s after photostress in a healthy volunteer (HV), in a migraine patient without aura (MO), with aura (MA) between attacks and in a patient during a migraine attack (MI).

All VEP recordings were found to be analyzable. VEP traces recorded before and after PS in selected HV, MO, MA, and MI patients are shown in Figure 1. The demographic and clinical features of recorded participants are shown in Table 1.

Before PS, the VEP P100 implicit time and N75-P100 amplitude did not differ significantly between groups (F(3,61) = 0.94, *p* = 0.43; F(3,61) = 0.54, *p* = 0.65, respectively).

**Figure 2:**
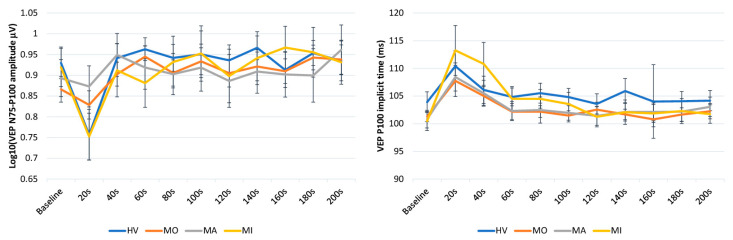
Graphic representation of mean ± SEM values of the N75-P100 VEP amplitude (left panel) and the P1 implicit time (right panel) at baseline and every 20 s after photostress up to 200 s in healthy volunteers (HV), in patients with migraine with aura (MA) and without aura (MO) between attacks, and in patients recorded during an attack (MI).

The ANOVA for P100 implicit time in averaged successive VEP blocks disclosed a main effect of time (F(10,610) = 11.472, *p* < 0.001), but not of groups (F(3,61) = 0.39, *p* = 0.76) and of the two-way interaction of group by time (F(30,610) = 0.95, *p* = 0.54) (Figure 2, right panel). The post hoc analysis showed that the 20 s VEP P100 implicit time was significantly delayed after PS as compared to baseline in all subject groups (all *p* < 0.001). The VEP P100 percentage changes at 20 s post PS of the baseline recording did not differ significantly between groups (F(2,54) = 0.42, *p* = 0.74).

**Figure 3:**
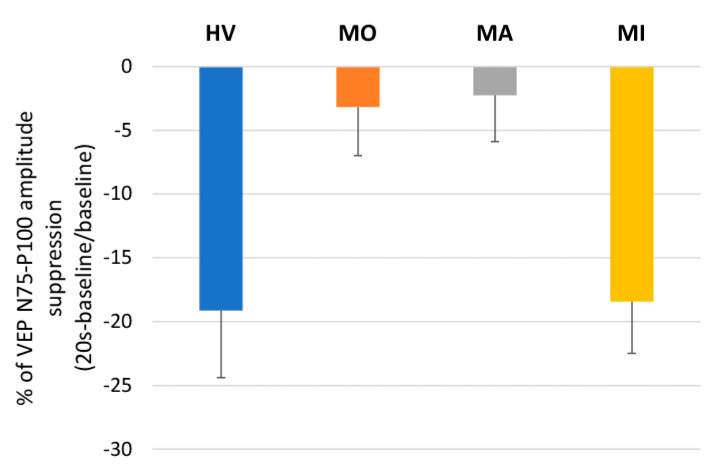
Mean ± SEM percentage changes from the baseline of visual evoked potentials (VEPs) for the N75-P100 amplitude at 20 s after photostress in healthy volunteers (HV), patients with migraine with aura (MA) and without aura (MO) between attacks, and in patients recorded during an attack (MI).

The ANOVA for the N75-P100 amplitude in averaged successive VEP blocks disclosed a main effect of time (F(10,610) = 12.31, *p* < 0.001) and of the two-way interaction of group by time (F(30,610) = 1.76, *p* = 0.008), but not of groups (F(3,61) = 0.03, *p* = 0.992) (Figure 2, left panel). The post hoc analysis showed that, compared to the baseline value, the VEP N75-P100 amplitude was significantly reduced immediately after PS in HV (*p* = 0.0001) and MI (*p* = 0.009), whereas it was nonsignificantly reduced both in MO (*p* = 1.00) and in MA (*p* = 1.00). Immediately after PS, the VEP N75-P100 percentage changes of the baseline recording differed significantly between groups (F(3,61) = 4.41, *p* = 0.007, Figure 3), resulting in less suppressed in MO (−3.18%, *p* = 0.031 vs. HV) and MA (−2.26%, *p* = 0.026 vs. HV) than in HV (−19.12%) and MI (−18.43%).

**Figure 4:**
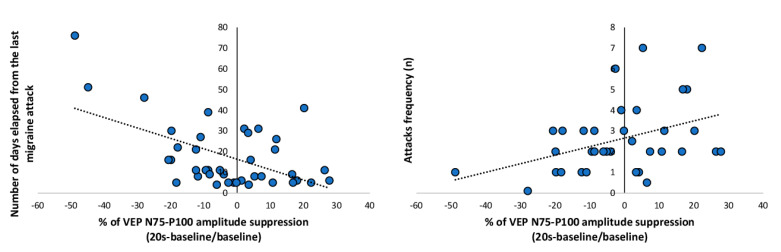
VEP percentage of the N75-P100 amplitude suppression from baseline recorded in patients with migraine between attacks (MA + MO) at 20 s after photostress plotted as a function of number of days elapsed from the last migraine attack (left panel) and attack frequency (right panel). The regression line is fitted to the data points.

When all patients recorded between attacks (MO, MA) were combined, the VEP 75-P100 percentage changes immediately after PS of the baseline recording was correlated negatively with the number of days since the last migraine attack (r = −0.547, *p* < 0.001, Figure 4 left panel), and positively with the attacks frequency (r = 0.402, *p* = 0.017, Figure 4 right panel).

No correlations were disclosed between neurophysiological parameters and duration of the migraine disease or monthly mean duration of migraine attacks.

## 4. Discussion

The most interesting result of our study was that the level of suppression of VEP amplitude after photostress was reduced in migraine patients between attacks, regardless of the presence of aura, but was normal during an attack. The fact that both patients with MO and MA behaved in the same way during the pain-free phase may indicate that the results of our study related more to migraine as such than to aura. Moreover, when patients with MO and MA were combined, VEP suppression was positively related to the days elapsed since the last attack and negatively related to the frequency of migraine attacks.

Photostress testing, in which the retina is bleached with light and recovery of its functionality is objectively measured with VEPs, is a well-established way to test the functional capability of the macula and its projections to the cortex [16]. The main factor determining the recovery time of normal vision and, therefore, of a normal amplitude of the evoked potentials, after PS is the resynthesis of visual pigments in the cones and rods of the retina. In fact, it is known from the animal model that at any level of visual adaptation, there is a certain balance between the rate of glare and the rate of regeneration of photopigments [27]. Therefore, while we cannot completely exclude a faster regeneration of photopigments, the fact that we did not observe delayed—a common finding in disorders clearly limited to the macular region [28]—but faster recovery of VEP amplitude in our patients with migraine, we concluded there was a negligible involvement of mechanisms of photopigment resynthesis in our VEP results. However, the presence of transient or persistent variations in the thickness of the retinal fiber layer and ganglion layer could be other causative factors that may have taken part in the process of VEP recovery after PS in our patients, despite inconclusive data from the literature on this topic [29,30,31].

It is well known that the effect of light is to dazzle the photopigments into a mixture of yellow and colorless intermediates [32]. This is remarkably interesting, since the visual discomfort frequently associated with migraine, often present even in the pain-free period, seems to be, at least in part, mediated by a specific yellow–blue non-image-forming pathway that conveys information from the retinal ganglion cells (RGCs) to the posterior thalamic nuclei and cortex [1,14]. Light discomfort associated with bleaching light could thus help explain our results of a lack of VEP amplitude suppression after PS, which in turn may be explained by RGCs less prone to suppress visual input. Whether this lack of suppression was related to the postretinal amplification of intrinsically photosensitive RGCs observed by others in relation to stimulation intensity [15], and whether it was the cause or the consequence of the lower threshold for light discomfort in migraine patients between attacks [33], remain to be determined. However, the fact that VEP suppression after photostress returns to normal during an attack, when photophobia is at its highest, argues against the involvement of a pathological mechanism located at the level of the ganglion cell alone, but opens an additional postganglionic involvement.

In fact, VEPs reflect the mass activity of the entire visual pathway. Interestingly, in patients with a definite diagnosis of multiple sclerosis, only those with a previous optic-nerve dysfunction showed VEP abnormalities after photostress, not those without this history [22]. This suggests that VEP recovery after PS depends on the integrity of the entire visual nerve pathway.

It is of interest that the information on illumination that is too intense and dazzling is transmitted from the retina ganglion to key thalamic and brainstem nuclei involved in the trigeminovascular pathway [14,34]. Thalamic nuclei in turn send visual information to somatosensory, visual, and associated brain areas [35]. Interestingly, a lesion at the thalamic level can increase dazzle photosensitivity [36].

That the thalamic hub between the retina and cortex may be at the origin of central dazzle is relevant to our study because an increasing amount of evidence suggests the presence of an abnormal crosstalk between the thalamus and cortex in migraine, especially between attacks [37,38]. The multiband rhythmic activity of the visual cortex under the direct control of the thalamic nuclei [39,40], in conjunction with the activation of peculiar inhibitory systems at the cortical level [2,41,42,43], are altered in migraine. This general thalamo-cortical network dysexcitability disallows the occurrence of physiological synaptic plasticity induced by the presentation of visual stimuli, which is in line with our present findings of lack of dazzling light-induced VEP inhibition. That an aberrant synaptic plasticity is at the base of behavioral VEP responses finds support in previous observations that neuromodulatory interventions such as experimentally induced visual deprivation [44], tonic pain [45], and transcranial paired associative stimulation [46] were also unable to modify VEP amplitudes in migraine, but did so in healthy controls.

All this evidence suggests that migraine is part of the spectrum of thalamocortical dysrhythmia syndromes [47], as also indicated by numerous morphofunctional neuroimaging studies [48,49,50,51,52,53].

In a further provocative finding, we observed that the lack of inhibition of the visual response after PS varied over time in a manner dependent on when the patient was recorded between attacks: the closer the patient was to the previous attack, the lower the percentage change of VEP amplitude after photostress. That the level of response to sensory stimulation, including visual stimulation, in migraine depends on the distance from the last attack has been seen several times before [2,41,54,55,56]. The same distance-dependent functional change since the attack has also been observed at the brainstem [57,58] and thalamic [48] levels, suggesting once more that alteration of synaptic plasticity mechanisms at multiple levels takes part in the pathophysiology of migraine attack recurrence. The lack of VEP inhibition reached the maximum just before an attack, when then the response reverted to normal. Recent neuroimaging findings indicate that a complex interplay between the hypothalamus and the trigemino-vascular system is engaged with proximity of the migraine attack, i.e., during the so-called premonitory phase of migraine [59,60,61]. These systems are designed to preserve brain homeostasis by regulating homeostatic needs [62]. We argue that the visual cortex’s increased energetic needs, due to its interictal progressively increased responsivity, can lead to a critical point when a mismatch between energetic reserve and demands may occur, and then the hypothalamo-trigeminovascular system ignites with a scope to re-establish a normal homeostatic balance [63]. Back to our study, this reflects in the re-establishing of a normal VEP amplitude suppression after PS, probably through both direct or indirect hypothalamic nuclei connectivity with the occipital cortex [64].

Nonetheless, we observed that the higher the migraine frequency, the more VEP amplitude suppression was lacking after PS. This is probably because patients with a high frequency of the disease have a shorter time from the previous attack. These data support the strong relationship between the severity of presentation of migraine disease and the behavior of neurophysiological responses.

That the levels of response to sensory stimulations, including visual stimulation, in migraine are not stationary, but dynamically change up to the tipping point of the attack, has been seen several times before [2,41,54,55,56], mimicking changes at the brainstem (dorsal pons [58] and trigeminal nuclei [57]) and thalamic [48] levels. This suggests once more that disease-related dysrhythmic thalamocortical activity disallows the occurrence of physiological response to photostress and might take part in the pathophysiology of migraine attack recurrence.

In the end, as a limitation of the study, we must recognize that, despite patients being recorded during the two typical periods of the migraine cycle, the patients in the two ictal/interictal groups did not serve as their own controls. This makes immediate generalization of the results impossible.

Finally, it remains necessary to verify whether these aberrant synaptic plasticity mechanisms in migraine, by reorganizing neural maps in the thalamocortical network, might consequently alter the interpretation of the information coming from the peripheral receptors. If this were the case, this could easily explain the subtle abnormalities in color perception suggested by numerous studies of migraine [8,9,10,11,12,13].

## Figures and Tables

**Table 1:** Demographic and clinical features of healthy volunteers (HV), migraine patients without aura (MO), with aura (MA) between attacks, and in patients with migraine during an attack (MI). Data are expressed as means ± SD or percentages (%).

Characteristics	HV (*n* = 14)	MO (*n* = 22)	MA (*n* = 19)	MI (*n* = 10)
Women (*n*)	10	16	12	8
Age (years)	29.7 ± 6.1	29.2 ± 8.2	30.3 ± 10.2	32.7 ± 11.7
Duration of migraine history (years)		17.3 ± 9.7	18.1 ± 11.5	15.6 ± 7.6
Attack frequency/month (*n*)		2.4 ± 1.6	2.5 ± 1.5	2.1 ± 1.8
Attack duration (hours)		33.2 ± 26.6	24.9 ± 26.5	40.7 ± 27.6
N° of days since the last attack		17.4 ± 17.2	14.4 ± 12.5	
Scintillating scotoma/Fortification spectra			100%	
Sensory symptoms			36.8%	
Language/Speech symptoms			15.8%	

## Data Availability

The informed consent form signed by all participants in this study did not include a provision stating that individual raw data can be made publicly accessible. Researchers meeting the criteria for access to confidential data may access the data upon request from the corresponding author.

## Abbreviations

HV	healthy volunteers
ICHD	international classification of headache disorders
MA	patients with migraine with aura between attacks
MI	patients with migraine recorded during an attack
MO	patients with migraine without aura between attacks
PS	photostress
VEP	visual evoked potential
